# Longitudinal co-variations between inflammatory cytokines, lung function and patient reported outcomes in patients with asthma

**DOI:** 10.1371/journal.pone.0185019

**Published:** 2017-09-15

**Authors:** Karin Lodin, Mats Lekander, Jörgen Syk, Kjell Alving, Predrag Petrovic, Anna Andreasson

**Affiliations:** 1 Department of Neurobiology, Care Sciences and Society, Division of Family Medicines and Primary Care, Karolinska Institutet, Huddinge, Sweden; 2 Department of Clinical Neuroscience, Karolinska Institutet, Stockholm, Sweden; 3 Stress Research Institute, Stockholm University, Sweden; 4 Centre for Allergy Research, Karolinska Institutet, Stockholm, Sweden; 5 Department of Women’s and Children’s health, Uppsala University, Uppsala, Sweden; 6 Academic primary health care centre, Stockholm County Council, Stockholm, Sweden; 7 Department of Psychology, Macquarie University, North Ryde, New South Wales, Australia; National and Kapodistrian University of Athens, GREECE

## Abstract

**Background:**

Asthma is a chronic inflammatory respiratory disorder associated with reduced lung function and poor quality of life. The condition is also associated with poor self-rated health, a major predictor of objective health trajectories. Of biological correlates to self-rated health, evidence suggests a role for inflammatory cytokines and related sickness behaviours. However, this is mainly based on cross-sectional data, and the relation has not been investigated in patients with chronic inflammatory conditions.

**Objective:**

To investigate inflammatory cytokines, lung function, sickness behaviour and asthma-related quality of life as determinants of self-rated health in patients with asthma, and to investigate if these variables co-vary over time.

**Methods:**

Plasma cytokines (IL-5, IL-6), lung function (FEV1), sickness behaviour, asthma-related quality of life and self-rated health were assessed in 181 patients with allergic asthma aged 18–64 years in a one-year longitudinal study. Mixed effect regression models and Spearman’s correlation were performed to analyse the associations between repeated measurements.

**Results:**

More sickness behaviour and poorer asthma-related quality of life were associated with poorer self-rated health (p’s<0.001). In men, both low and high levels of interleukin (IL)-6 and poorer lung function were related with poorer self-rated health (p’s<0.05). Over the year, improved asthma-related quality of life was associated with better self-rated health (Spearman’s rho = -0.34 women,-0.36 men, p’s<0.01). Further, if sickness behaviour decreased, self-rated health improved, but only in women (Rho = -0.21, p<0.05). Increased FEV1 in men was associated with an increase in IL-6 (Rho = 0.24, p<0.05) as well as improved self-rated health (Rho = -0.21, p<0.05) and asthma-related quality of life (Rho = 0.29, p<0.01) over the year.

**Conclusion:**

The study highlights the importance of subjectively perceived sickness behaviour and asthma-related quality of life together with lung function as determinants of self-rated health in asthmatic patients. The importance of inflammatory activation for patient reported outcomes in chronic inflammatory conditions need further investigation.

## Introduction

Patient reported outcomes (PROMs) such as self-rated health and quality of life, reported directly by the patients without interpretation of anyone else, are becoming increasingly used in clinical practice. Self-rated health equals and in many cases surpasses objective measures in predicting objective long-term health outcomes such as mortality and morbidity [1–3]. Poor self-rated health and poor quality of life have been associated with higher levels of pro-inflammatory cytokines such as interleukin (IL)-6 and tumour necrosis factor (TNF)-alpha in general populations and primary care patients [4–7]. However, the relation between such central PROMs and pro-inflammatory cytokines in patients with chronic inflammatory conditions, such as asthma, is unknown.

Allergic asthma is a chronic inflammatory respiratory disorder characterized by airway hyper responsiveness, increased mucus secretion and airway remodelling resulting in airway obstruction and reduced lung function [8]. The Th2-driven inflammatory response characteristic in asthma is driven by different inflammatory cytokines. For instance, IL-5 and IL-6 are involved in evoking systemic IgE responses and local eosinophil accumulation [9–11]. Asthma is further associated with poor self-rated health [12] and poor asthma-related quality of life in a way which cannot fully be explained by objective measures of reduced lung function [13]. Indeed, correlations between for instance ratings of subjective breathlessness, and objective clinical measures of lung function such as forced expiratory volume during one second (FEV1) are generally weak but significant [13–18]. Patient reported outcomes have further been shown to be able to improve even in absence of objective improvement in lung function [18]. This discrepancy between subjective patient reported outcomes and objective measures of lung function in patients with asthma is in accordance with the biopsychosocial model introduced by Engel in 1977, which suggest that disease burden cannot be inferred by biomedical data only, but needs to also reflect psychological and social processes [19].

Building on the inflammatory etiology of asthma and on designated immune-to-brain pathways, inflammatory cytokines and cytokine-induced sickness behaviour are factors of interest as determinants for poor self-rated health in patients with asthma. Inflammatory cytokines are known to influence the brain acting through the interoceptive pathway [20–22]. It is suggested that inflammatory cytokines are involved as markers of bodily condition when overall health is appraised by an individual. This is based on neurophysiological findings [23] and the fact that the immune-to-brain signalling during inflammation leads to a co-ordinated set of changes called sickness behaviour [24–30]. In an acute setting, sickness behaviour promotes recovery, but if the inflammation persists and becomes chronic, it is thought to contribute to ill-health [24] and poor perceived health [6, 23]. Sickness behaviour includes symptoms such as fatigue, increased pain sensitivity, lack of energy, malaise, anorexia and anhedonia [31], resembling key determinants of poor self-rated health [6, 32]. Therefore, cytokine-induced sickness behaviour has been suggested to be an important pathway to suboptimal self-rated health [6, 28].

We have previously reported that elevated levels of inflammatory cytokines and sickness behaviour are important determinants of self-rated health in primary health care populations [6, 32]. However, it is not yet known if asthma-related cytokines and asthma-related quality of life contribute to poor self-rated health in patients with a chronic inflammatory condition such as asthma. Furthermore, since most previous studies have been cross-sectional, it is not known if changes in cytokines or in other clinical measures are followed by changes in self-rated health, findings which would strengthen suppositions of causal relationships between the variables. We hypothesised that impaired lung function and increased levels of inflammatory cytokines due to chronic allergic asthma would be associated with increased sickness behaviour and poor asthma-related quality of life, which would all have a negative impact on self-rated health.

The aim of the present study was to investigate two relevant cytokines, IL-5 and IL-6, FEV1 (% predicted), sickness behaviour and asthma-related quality of life as determinants of self-rated health in patients with asthma. In addition, to investigate the co-variation over time between inflammatory cytokines and subjective patient reported outcomes (sickness behaviour, self-rated health, asthma-related quality of life) in relation to objective clinical measures in this one-year longitudinal study.

## Material and methods

### Participants

The NOAK-study (Optimization of the anti-inflammatory treatment of asthma through exhaled nitric oxide for increased asthma-related quality of life) is a longitudinal randomized controlled trial on asthma symptom control, asthma-related quality of life and fraction of exhaled nitric oxide (F_E_NO)-guided treatment [33]. A brief description of methods and measures relevant to the present study is given here. For details, see elsewhere [33]. All participants gave written informed consent. The study was approved by the regional ethics committee in Stockholm (Dnr: 2006/185-31) and registered in Clinical Trials, NCT00421018.

Patients were recruited from 17 primary health care centres in seven different county councils in central and southern Sweden from November 2006 to March 2010. In total, 181 patients with asthma who had confirmed IgE sensitization to at least one airborne perennial allergen (87 women, 94 men) aged 18 to 64 years, were included. All participants were non-smokers since at least one year before inclusion and had a previous smoking history of maximum 10 pack-years. In addition, eligible patients all had a physician’s diagnosis of asthma and had been on a medication with inhaled corticosteroids (ICS) since at least 6 months before the inclusion.

### Procedure

Participants being treated with combination inhalers (corticosteroids plus long-acting beta-2-agonists) had to withdraw the long-acting beta-2-agonist and switch to two separate inhalators with the corresponding dosage of short acting beta-2 agonist and corticosteroid. The patients were randomised in a straight randomisation by lottery into two groups. In the F_E_NO-guided treatment group (n = 93) the anti-inflammatory treatment (inhaled corticosteroids (ICS) and leukotriene-receptor antagonist (LTRA; montelukast 10 mg daily) was adjusted on the basis of F_E_NO. In the control group (n = 88), treatment was adjusted based on symptoms according to routine clinical practice [33]. Antihistamines, local steroids and sodium chromoglycate were allowed for treatment of rhinitis and conjunctivitis. However, treatment with cortisone injections was not allowed.

In total, the study included six visits at the health care center for the participants: visit 0 (inclusion, -2-4 weeks), visit 1 (0 months), visit 2 (2 months), visit 3 (4 months), visit 4 (8 months) and visit 5 (12 months). SRH was measured at each visit, and sickness behaviour and mAQLQ at visit 1, 3 and 5. Venous blood for analysis of levels of cytokines was sampled in EDTA tubes at visit 1 and 5. The samples were centrifuged and initially stored at -20°C before aliquots of plasma were transferred to -70°C until analysis.

### Measures

#### Inflammatory cytokines

Circulating levels of interleukin (IL)-5 and IL-6 were analysed from plasma samples with OLINK multiplex immunoassay (Olink Proteomics, Uppsala, Sweden). Minimal detectable concentrations for IL-5 were 3.81 pg/mL and 3.01 pg/ml for IL-6. Each patient’s samples, from baseline and 12 months, were analysed side-by-side in the instrument for all measurements. For IL-5, 87 values from visit 1 and 92 values from visit 5 were below the detection limit and were set to the lowest detectable concentration. All IL-6 values were above the detection limit. Blood samples were missing for 22 participants. All cytokine values were z-transformed prior to analysis to facilitate interpretation of regression coefficients (b).

#### Lung function

Lung function measured as forced expiratory volume in one second (FEV1) was assessed through spirometry (Vitalograph, Spirotrac IV, Buckingham, England) at visit 1 and 5. Percent of predicted FEV1 was calculated using Hedenström’s reference values.

#### Sickness behaviour

A composite measure similar to that used in Undén et al. 2007 was used to assess sickness behaviour. The composite measure included weighted means of the answers to the questions “how satisfied are you with your situation regarding the following aspects: energy/sleep/fitness/appetite and memory”. The responses on each item were rated on a Likert scale ranging from “very poor” (1) to “excellent, could not be better” (7). The scale was inverted in order to facilitate interpretation. Thus, a higher score corresponds to a higher degree of sickness behaviour.

#### Self-rated health

Self-rated health was measured using the question “How would you rate your general health status” The response alternatives were: Very good (1), Rather good (2), Neither good nor poor (3), Quite poor (4) and Poor (5).

#### Asthma-related quality of life

Asthma-related quality of life was measured using the Mini-AQLQ questionnaire (mAQLQ) consisting of 15 items aimed to measure functional problems (physical, emotional, social and occupational) that are most troublesome to adults with asthma [34]. The responses on each item were rated on a Likert scale ranging from “all of the time/ totally limited” (1) to “none of the time/ not at all limited” (7).

#### Background factors

Height and weight were measured at the first visit and used to calculate body mass index (kg/m^2^).

### Statistics

Study group characteristics are presented separated on gender and differences between genders were tested using Student’s t-test or Mann-Whitney U-test when applicable. Because data were missing for some observations for a few participants Ns vary slightly between analyses.

First, mixed effect regression analysis using identity as random effect with time as a dummy variable was used to test if self-rated health, sickness behaviour, asthma-related quality of life, inflammatory cytokines or FEV1 (% predicted) changed during the course of the study. Values from visit 1 (baseline) and visit 5 (one-year follow-up) were included in the regression model.

Second, the overall associations between the same variables were calculated using mixed effect regression analyses. IL-5 and IL-6 were analysed separately, and models were stratified for sex. All models were adjusted for age and BMI and included all available data points. Corticosteroid dose and treatment with LTRA were included in a follow-up analysis of any found significant associations to test anti-inflammatory treatment as a confounder. Due to the non-normal properties of the included variables the p-values were estimated by bootstrap with 1000 repetitions [35]. A univariate cubic regression spline model with three degrees of freedom and an alpha-level of 0.05 was used explorative to test for linearity in the associations between IL-5 and IL-6 and patient reported outcomes [36].

Third, to investigate if changes in self-rated health were associated with a change in sickness behaviour, asthma-related quality of life, inflammatory cytokines and FEV1 (% predicted), the change in self-rated health, sickness behaviour, asthma-related quality of life inflammatory cytokines and FEV1 (% predicted) from visit 1 to visit 5 was calculated and the delta values were correlated. Due to the non-normal properties of the delta-values, Spearman rank correlations were used.

STATA^®^ 14.0 (StataCorp, LP, Texas, USA) were used for all analyses. An α-level of 0.05 was used to test for significance.

## Results

### Study group characteristics

Baseline characteristics for the participants are presented in Table 1. Half of the participants were women (48%) and the average age was 41 years. The participants reported good asthma-related quality of life (initially rated as 5.7 for women and 5.8 for men on a seven point scale where higher ratings correspond to better asthma-related quality of life). The participants reported on average “rather good” self-rated health (2.1 in women and 2.0 in men on a five point scale). There were no significant differences between women and men at baseline or one-year follow-up regarding any of the investigated parameters age, BMI, inflammatory cytokines, FEV1, sickness behaviour, self-rated health and asthma-related quality of life (p-values are reported in Table 1)

**Table 1:** Demographic factors, self-rated health, sickness behaviour and inflammatory markers in men and women at baseline and one-year follow-up.

	Women				Men				
	n	range	mean	SD	n	range	mean	SD	p
*baseline*									
Age	87	19–63	41.4	11.9	94	18–64	40.6	12.8	0.68^†^
BMI	83	17.6–45.2	26.7	6.0	93	18.7–39.4	26.5	3.8	0.72^†^
IL-5	76	3.6–43.3	6.3	7.7	83	3.6–47.4	5.7	7.2	0.63^†^
IL-6	76	4.1–180.5	12.9	20.7	83	4.1–49.2	10.0	6.6	0.22^†^
FEV1^1^	84	50.9–119.6	82.7	13.9	86	56.1–105.5	84.2	12.7	0.20
Sickness behaviour^2^	85	1.0–5.8	3.1	1.0	93	1.2–5.2	3.0	0.9	0.96^††^
Self-rated health^3^	86	1.0–5.0	2.1	0.8	93	1.0–4.0	2.0	0.8	0.65^††^
mAQLQ^4^	82	3.0–6.9	5.7	0.9	92	3.1–7.0	5.8	0.9	0.19^††^
*one-year follow-up*									
IL-5	76	3.6–30.3	5.6	5.2	83	3.6–28.7	4.9	4.0	
IL-6	76	3.9–184.6	13.1	20.8	83	4.0–953.1	22.4	104.4	0.45^†^
FEV1^1^	79	49.6–118.2	82.4	13.1	86	56.1–105.5	84.2	12.7	0.36^†^
Sickness behaviour^2^	80	1.0–5.8	3.0	1.0	84	1.4–5.2	2.9	0.7	0.78^††^
Self-rated health^3^	80	1.0–5.0	2.0	0.8	84	1.0–4.0	1.9	0.7	0.93^††^
mAqlq^4^	76	2.0–7.0	5.9	0.9	84	3.0–7.0	6.1	0.8	0.12^††^

^1^Higher FEV1 denotes better lung function. Values are reported as FEV1 percent predicted

^2^Composite variable of rating of energy, sleep, memory, fitness and appetite where higher scores denote more pronounced sickness behaviour

^3^Higher scores on self-rated health denote worse self-rated health

^4^Higher scores denotes better asthma-related quality of life

^†^Student’s t-test

^††^Mann-Whitney U test

Abbreviations: BMI—body mass index, IL—interleukin, mAqlq—mini asthma-related quality of life questionnaire, FEV1—forced expiratory volume in 1 second (% predicted)

Neither IL-6 (b = 6.53, 95%CI:-4.79;17.90, p = 0.26), FEV1 (% predicted) (b = -0.60, 95%CI:-1.68;0.48, p = 0.28), nor self-rated health (b = -0.09, 95%CI:-0.21;0.03, p = 0.13) changed significantly between baseline and follow-up. However, there was a significant decrease in IL-5 (b = -0.74, 95%CI:-1.39;-0.09, p = 0.03), an increase in asthma-related quality of life over time (b = 0.27, 95%CI:0.15;0.40, p<0.001) and a reduction in sickness behaviour (b = -0.10, 95%CI:-0.20;0.00, p = 0.04).

### Overall associations between subjective health ratings, lung function and inflammatory cytokines

**Fig 1:**
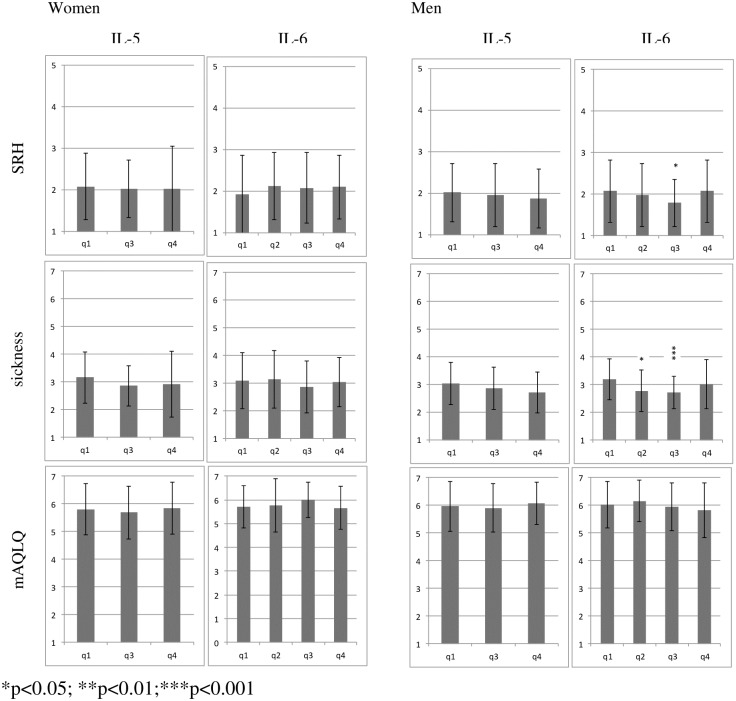
Distribution of self-rated health, sickness behaviour and asthma-related quality of life per cytokine-quartiles. Higher scores on the y-axis denote worse self-rated health, more pronounced sickness behaviour and better asthma-related quality of life. Error bars are used for standard deviation. For IL-5, quartile one and two were collapsed to one reference category due to a substantial proportion of IL-5 falling below the limit of detection. Abbreviations: SRH—self-rated health, IL—interleukin, mAQLQ—mini asthma-related quality of life questionnaire, q—quartile.

As the associations between cytokines and patient reported outcomes were found to be non-linear in the cubic spline regression analyses, IL-5 and IL-6 were divided into quartiles and the lowest category was used as reference in the analyses. Table 2 shows the overall associations between sickness behaviour, asthma-related quality of life, self-rated health and inflammatory cytokines divided into quartiles with quartile 1 being reference. Because a substantial proportion of IL-5 (55% at visit 1 and 58% at visit 5) fell below the limit of detection, quartile one and two were collapsed to one reference category. The associations between the PROMs and the inflammatory cytokines, divided into quartiles are presented in Fig 1.

**Table 2:** Associations between sickness behaviour, mAQLQ, self-rated health and inflammatory cytokines reported in quartiles with quartile 1 as reference.

	Women Sickness^1^	mAQLQ^2^	SRH^3^	Men Sickness^1^	mAQLQ^2^	SRH^3^
IL-5						
quartile 3	-0.02	-0.16	0.00	-0.07	-0.08	0.02
quartile 4	-0.27	-0.07	0.08	-0.22	0.15	-0.11
IL-6						
quartile 2	0.01	-0.02	0.22	-0.36*	0.07	-0.24
quartile 3	-0.03	0.13	0.21	-0.54***	-0.05	-0.37*
quartile 4	0.04	-0.33	0.25	-0.26	-0.28	-0.18

*p<0.05;

**p<0.01;

***p<0.001

^1^Composite variable of rating of energy, sleep, memory, fitness and appetite where higher scores denote more pronounced sickness behaviour

^2^Higher scores denotes better asthma-related quality of life

^3^Higher scores on self-rated health denote worse self-rated health

The cytokines values were z-transformed to facilitate interpretation of b-coefficients. All models were adjusted for age and BMI and included all available data points.

No associations were found between IL-5 and any of the patient reported outcomes. Men belonging to middle quartiles of IL-6 reported significantly better self-rated health (quartile 3) and significantly lower sickness behaviour (quartile 2 and 3) compared to the reference quartile 1. Given this pattern, we explored if there were u-shaped relationships between IL-6 and self-rated health or sickness behaviour in men. In order to test if quartile 2 and 3 differed significantly from quartile 4 the reference categories were switched so that quartile 4 replaced quartile 1 as reference. Men belonging to quartile 3 in IL-6 reported significantly less sickness behaviour compared to quartile 4 (b = -0.26 p = 0.048). No significant associations were found for quartile 2 (b = -0.06 p = 0.67) and 1 (b = 0.26, p = 0.11) when quartile four was the reference quartile. Thus, the association was u-shaped where men belonging to the third quartile of IL-6 reported less sickness behaviour compared to men belonging to either the lowest or the highest quartile. There were no u-shaped association between levels of IL-6 and self-rated health (p’s>0.22). In women, no associations were found between cytokines and self-ratings.

**Fig 2:**
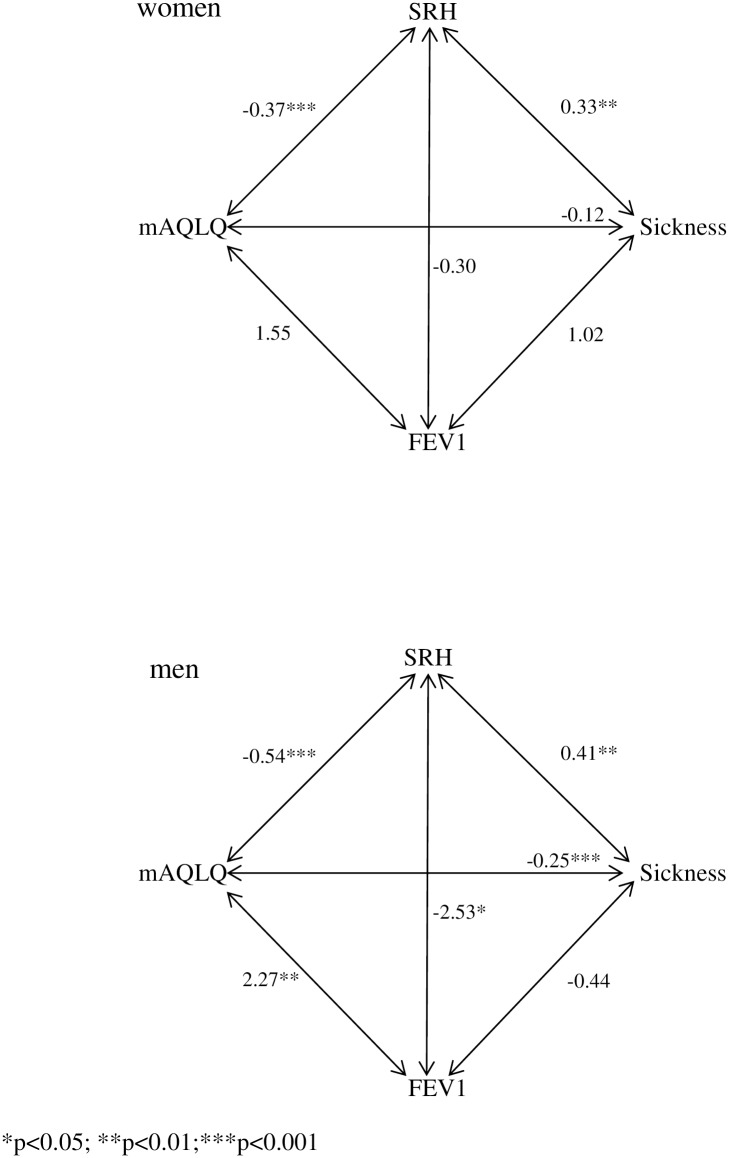
Overview of the associations between lung function, self-rated health, sickness behaviour and asthma-related quality of life in women and men presented as b-coefficients from the mixed regression analysis. Abbreviations: SRH—self-rated health, mAQLQ—mini asthma-related quality of life questionnaire, FEV1—forced expiratory volume in one second (% predicted).

Associations between lung function and patient reported outcomes (sickness behaviour, self-rated health and asthma-related quality of life) are presented numerically in Table 3 and schematically in Fig 2.

**Table 3:** Associations between lung function and patient reported outcomes.

	time-pts	obs	b women	CI	obs	b men	CI
*FEV1*^1^							
Sickness^2^	2	158	1.02	-0.94;2.98	176	-0.44	-3.06;2.19
SRH^3^	2	159	-0.30	-3.09;2.49	176	-2.53*	-4.64;0.43
mAQLQ^4^	2	151	1.55	-0.93;4.02	175	2.27**	0.62;3.92
*Sickness*							
SRH	3	237	0.33***	0.18;0.48	262	0.41***	0.23;0.58
mAQLQ	3	228	-0.12	-0.25;0.01	259	-0.25***	-0.38;-0.12
*SRH*							
mAQLQ	3	227	-0.37***	-0.53;-0.19	259	-0.54***	-0.73;-0.34

*p<0.05;

**p<0.01;

***p<0.001

^1^Higher FEV1 denotes better lung function. Values are reported as FEV1 percent predicted

^2^Composite variable of rating of energy, sleep, memory, fitness and appetite where higher scores denotes more pronounced sickness behaviour

^3^Higher scores on self-rated health denote worse self-rated health

^4^Higher scores denotes better asthma-related quality of life

Fixed effect coefficients (b) and 95% confidence intervals (bootstrapped based p-values) for the association between self-rated health, inflammatory cytokines, sickness behaviour, asthma-related quality of life and FEV1. All models were adjusted for age and BMI and included all available data points.

Abbreviations: Time-pts—number of time points included in analysis, obs—number of observations in analysis, b—beta coefficient, CI—confidence intervals, IL—interleukin, FEV1—forced expiratory volume in one second (% predicted)

Better lung function was significantly associated with better self-rated health and better asthma-related quality of life in men. In women, there were no associations between lung function and any of the patient reported outcomes.

High ratings of sickness behaviour and poor asthma-related quality of life were significantly associated with poor self-rated health in both men and women. Furthermore, high ratings on sickness behaviour were associated with poor asthma-related quality of life in men but not in women.

We tested if adjustment for anti-inflammatory treatment would influence the strength of any of the above mentioned significant associations. The b-coefficient for any of the associations between cytokines, lung function, sickness behaviour, asthma-related quality of life and self-rated health changed with less than 5% after including corticosteroids or LTRA treatment, respectively, in the model, apart from the association between quartile 4 compared to quartile 1 of IL-6 and sickness behaviour, which was strengthened by 16% after including ICS treatment. Hence, anti-inflammatory treatment did not explain the association between cytokines or lung function and patient reported outcomes.

### Longitudinal associations between changes in inflammatory cytokines, patient reported outcomes and lung function

The associations between changes in inflammatory cytokines, patient reported outcomes and lung function are presented in Tables 4 and 5. In women, both a decrease in sickness behaviour and an increase of asthma-related quality of life was associated with an improvement in self-rated health over time.

**Table 4:** Spearman’s correlations for changes in cytokines, FEV1, sickness behaviour, asthma-related quality of life and self-rated health between baseline and follow-up in women.

Women	IL-5	IL-6	FEV1	Sickness	SRH	mAQLQ
IL-5	1.0000					
IL-6	-0.0557	1.0000				
FEV1^1^	-0.0748	0.0235	1.0000			
Sickness^2^	0.0507	0.0651	-0.0616	1.0000		
SRH^3^	-0.1839	-0.0384	-0.0875	-0.2122*	1.0000	
mAQLQ^4^	0.0369	0.0512	0.0701	0.0479	-0.3378**	1.0000

*p<0.05;

**p<0.01;

***p<0.001

^1^ Percent predicted

^2^Composite variable of rating of energy, sleep, memory, fitness and appetite where higher scores denotes more pronounced sickness behaviour

^3^Higher scores on self-rated health denote worse self-rated health

^4^Higher scores denotes better asthma-related quality of life

**Table 5:** Spearman’s correlations for changes in cytokines, FEV1, sickness behaviour, asthma-related quality of life and self-rated health between baseline and follow-up in men.

Men	IL-5	IL-6	FEV1	Sickness	SRH	mAQLQ
IL-5	1.0000					
IL-6	0.0815	1.0000				
FEV1^1^	0.0146	0.2440*	1.0000			
Sickness^2^	-0.1366	-0.1217	-0.0073	1.0000		
SRH^3^	0.1060	0.0413	-0.2147*	-0.1680	1.0000	
mAQLQ^4^	-0.0778	0.1336	0.2903**	0.1052	-0.3579**	1.0000

*p<0.05;

**p<0.01;

***p<0.001

^1^ Percent predicted

^2^Composite variable of rating of energy, sleep, memory, fitness and appetite where higher scores denotes more pronounced sickness behaviour

^3^Higher scores on self-rated health denote worse self-rated health

^4^Higher scores denotes better asthma-related quality of life

In men, improved lung function was associated with increased levels of IL-6, increased asthma-related quality of life and improved self-rated health. Further, an increase in asthma-related quality of life was associated with improvement in self-rated health. In women, no associations were found between changes in lung function and changes in patient reported outcomes. There were no correlations between changes in inflammatory cytokines and patient reported outcomes in either men or women.

## Discussion

In this study, inflammatory cytokines, lung function, sickness behaviour and asthma-related quality of life were investigated as determinants of self-rated health in patients with chronic allergic asthma over a one-year period. We hypothesised that impaired lung function and increased levels of inflammatory cytokines due to chronic allergic asthma would be associated to increased sickness behaviour and poor asthma-related quality of life, which would all have a negative impact on self-rated health.

In consonance with the hypothesis, both sickness behaviour and asthma-related quality of life were significant determinants of self-rated health in patients with asthma. In addition, impaired lung function and increased mid-range levels of the pro-inflammatory cytokine IL-6 were found to be determinants of self-rated health in men.

An association between increased levels of inflammatory cytokines and poor self-rated health would have been expected according to our hypothesis, mainly based on previous findings in subjects not suffering from chronic inflammatory disorders [6, 32, 37]. In the present study, linear associations were not confirmed and men with either lower or higher levels of IL-6 were found to have poor self-rated health. The mid-range cytokine levels of IL-6 were associated with better self-rated health and asthma-related quality of life in men. Previously we have reported that either low [37] or high levels [6] of the pro-inflammatory cytokine IL-1β have been observed to be associated with poor subjective health. The role of inflammatory cytokines in physiological processes such as regulation of sleep [38] or memory consolidation [39] has been discussed. For instance, Barrientos et al. showed in a study in rats that long term potentiation of the memory is significantly impaired when levels of IL-1β are either too high or too low [39]. Therefore it cannot be excluded that certain levels of pro-inflammatory cytokines such as IL-6 are needed in order to maintain certain physiological processes, thereby contributing to a curvilinear relation between a pro-inflammatory cytokine and self-rated health.

Contrary to expectations, no association was found between inflammatory cytokines and any of the subjective health ratings in women. In previous research the link between inflammation and self-rated health was suggested to be more robust for women compared to men [6, 32, 37, 40, 41]. However, only a limited number of studies on self-rated health and inflammation have been stratified for sex and the sample sizes have generally been small with low statistical power and few men have been included [6, 42]. The lack of associations between inflammatory factors and subjective health in men in some studies should therefore be interpreted with caution. In one large study including 43 110 Swedish men aged 18–21 years, higher erythrocyte sedimentation rate indicative of low-grade inflammation was associated with poorer self-rated health, suggesting a link between subjective health and inflammation in healthy men [43].

There were significant differences between men and women regarding the association between objective measure of lung function and patient reported outcomes. In this study, an association between FEV1 (% predicted) and self-rated health was only found in men. No associations between any of the patient reported outcomes and objective measures were found in women. A number of studies have previously described the discrepancy between patient reported outcomes and objective clinical asthma measures. In a study by Ehrs and co-workers based on a Swedish primary health care population, no correlation was found between asthma-related quality of life and clinical measures of asthma such as lung function [44]. However, only 23 men (of 77 participants) were included in the study and it is possible that the analysis had too low power to detect any associations in men. Wechsler et al. conducted a study where 46 patients (80% women) with asthma where randomly assigned in a double-blind fashion to treatment with an albuterol inhaler, a placebo inhaler, sham acupuncture, or no intervention [18]. Although objective improvement did not significantly differ between the groups, the subjective improvement reported by the patients significantly improved with all three of the interventions compared to the control group. In a large study of almost 500 000 UK Biobank participants by Ganna et al. self-reported health was found to be the strongest predictor of all-cause mortality in men, but not in women where a previous cancer diagnosis was the strongest predictor [2]. Ganna further showed that FEV1 was one of the two strongest predictors among the physical measures of all-cause mortality. This finding, taken together with our results, could suggest that women and men include different aspects in the concept of self-rated health, with men perhaps including physical measures of importance for mortality prediction to a larger degree than women.

In the present study, higher sickness behaviour was strongly associated with poorer self-rated health over time, as hypothesised. This finding is in consonance with an earlier cross-sectional study of primary care patients where self-rated health was associated with a similar composite variable of sickness behaviour [32]. Importantly, our results shows for the first time in a longitudinal study that this association remains over time with an increase in sickness behaviour being associated with a decrease in self-rated health in both men and women.

The neural mechanisms linking objective measures (such as lung function or inflammatory cytokines) with subjective experiences (such as health or sickness ratings) are largely unknown. Of relevance, it has been hypothesised that the posterior and anterior parts of the insula play different roles in the objective-subjective trajectory [45–48]. According to this hypothesis, the posterior insula corresponds to a primary sensory cortex which processes interoceptive input, while the anterior part corresponds to an associative cortex, which processes higher order aspects like individual stimulus significance and subjective experience [45]. Consequently, aspects such as short- and long-term expectations have a greater impact on anterior as compared to posterior insula processing [49, 50]. It has been shown that acute experimental inflammation by injection of lipopolysaccharide in humans induces sickness behaviour and increases the functional connectivity between left anterior insula and middle cingulate cortex [21]. Partly in agreement with this model, objective symptoms of poor lung function have been connected with activation of the mid-insular and cingulate [46, 51]. In addition, Rosenkranz and co-workers reported that asthmatic patients under chronic stress, compared to a control group with asthmatic patients with low chronic stress, had increased activity in the mid insula in response to acute stress. This activity was associated with a greater airway inflammation and stress-induced increase in pro-inflammatory cytokine mRNA expression in the airways [52]. Thus, the insula has been suggested to have a central role in processing both objective aspects of asthma such as inflammation and symptoms of reduced lung function (posterior and mid-insula) as well as the subjective sickness related information (anterior insula).

This study has several strengths. First, this is the first longitudinal, multicentre study investigating co-variation over time between inflammatory cytokines and patient reported outcomes in relation to objective clinical measures in patients with asthma. Second, the patient sample consisted of a largely homogenous group of patients with well-managed mild to moderate allergic asthma representative for patients with asthma treated in primary health care in Sweden [33]. Even though all analyses were adjusted for LTRA and corticosteroid treatment, the present findings should still be interpreted with caution because all patients were on continuous anti-inflammatory treatment. In addition, patients with severe asthma were excluded, which reduced the possibilities to find prominent associations between the investigated variables. On a related note, a substantial proportion of IL-5 fell below the limit of detection, which reduced variability and statistical power. Another limitation is the use of a composite variable of sickness behaviour which does not cover all relevant aspects, such as pain and negative affect. A more comprehensive measure of sickness behaviour like the Sickness Questionnaire [53] could therefore be useful in future studies to better delineate behavioural factors that co-vary with inflammation and self-rated health in patients with asthma.

In conclusion, this study suggests sickness behaviour and asthma-related quality of life as determinants of self-rated health in patients with asthma. Furthermore, either too low or too high levels of IL-6 were, together with poor lung function, related to poor self-rated health in men in the present sample. Importantly, the study corroborates the importance of sickness behaviour and asthma-related quality of life as determinants of self-rated health by showing that such patient-reported outcomes co-vary over a one-year period in individuals suffering from allergic asthma.

## Supporting information

S1 Dataset Original data set. (XLSX)
