# Correction: Longitudinal co-variations between inflammatory cytokines, lung function and patient reported outcomes in patients with asthma

**DOI:** 10.1371/journal.pone.0197795

**Published:** 2018-05-17

**Authors:** Karin Lodin, Mats Lekander, Jörgen Syk, Kjell Alving, Predrag Petrovic, Anna Andreasson

In Tables 4 and 5, the direction of all correlations involving the variable *sickness* are reversed (i.e. positive correlations were labelled negative with a minus sign and vice versa). The correlations are described correctly in Results and the conclusion of the paper is not affected. Please see the corrected Tables 4 and 5 here.

**Table 4:** Spearman’s correlations for changes in cytokines, FEV1, sickness behaviour, asthma-related quality of life and self-rated health between baseline and follow-up in women.

Women	IL-5	IL-6	FEV1	Sickness	SRH	mAQLQ
IL-5	1.00					
IL-6	-0.06	1.00				
FEV1^1^	-0.07	0.02	1.00			
Sickness^2^	-0.05	-0.07	0.06	1.00		
SRH^3^	-0.18	-0.04	-0.09	0.21*	1.00	
mAQLQ^4^	0.04	0.05	0.07	-0.05	-0.34**	1.00

*p<0.05;

**p<0.01;

***p<0.001

^1^ Percent predicted

^2^Composite variable of rating of energy, sleep, memory, fitness and appetite where higher scores denotes more pronounced sickness behaviour

^3^Higher scores on self-rated health denote worse self-rated health

^4^Higher scores denotes better asthma-related quality of life

**Table 5:** Spearman’s correlations for changes in cytokines, FEV1, sickness behaviour, asthma-related quality of life and self-rated health between baseline and follow-up in men.

Men	IL-5	IL-6	FEV1	Sickness	SRH	mAQLQ
IL-5	1.00					
IL-6	0.08	1.00				
FEV1^1^	0.01	0.24*	1.00			
Sickness^2^	0.14	0.12	0.01	1.00		
SRH^3^	0.11	0.04	-0.21*	0.17	1.00	
mAQLQ^4^	-0.08	0.13	0.29**	-0.11	-0.36**	1.00

*p<0.05;

**p<0.01;

***p<0.001

^1^ Percent predicted

^2^Composite variable of rating of energy, sleep, memory, fitness and appetite where higher scores denotes more pronounced sickness behaviour

^3^Higher scores on self-rated health denote worse self-rated health

^4^Higher scores denotes better asthma-related quality of
